# Exposed embolization coils left in situ after embolization of a pseudoaneurysm inside walled-off pancreatic necrosis and subsequent successful direct endoscopic necrosectomy

**DOI:** 10.1055/a-2549-3220

**Published:** 2025-03-20

**Authors:** Yoshihisa Fukuda, Takamitsu Sakamoto, Shintaro Ryu, Kazuyuki Akiyama, Yukako Yagi, Koji Nakamichi, Tomoaki Noritomi

**Affiliations:** 1 38067 Gastroenterology, Fukuoka Tokushukai Hospital, Kasuga, Japan

Direct endoscopic necrosectomy (DEN) has become a standard and effective therapeutic option for walled-off pancreatic necrosis (WON); however, it can cause lethal bleeding from an intra-WON artery. Prophylactic coil embolization of the artery within the WON has been reported to prevent fatal bleeding during DEN 1 2 . Nevertheless, there has been no consensus on how long after embolization DEN can be safely performed without the risk of bleeding, or whether embolization coils in the WON should be removed. Herein, we describe a rare case of successfully treated WON, with embolization coils that remained in the WON space for 5 years post-DEN ( Video 1 ).

Direct endoscopic necrosectomy (DEN) is performed in a patient who was found to have a gastroduodenal artery pseudoaneurysm inside the walled-off necrosis (WON) and underwent embolization of the gastroduodenal artery; the metal embolization coils were found to be exposed inside the WON during DEN conducted 71 days after the embolization, with the decision made to leave the adherent coils in situ owing to the risk of bleeding.Video 1

A 64-year-old man with a 1-month history of severe necrotizing gallstone pancreatitis was transferred to our hospital for treatment of WON. On admission, a contrast-enhanced computed tomography (CE-CT) demonstrated the gastroduodenal artery coursing into the WON ( Fig. 1 **a** ). To provide drainage, two plastic stents were placed transgastrically into the WON, under endoscopic ultrasound guidance. A CE-CT performed 2 days later revealed a gastroduodenal artery pseudoaneurysm inside the WON ( Fig. 1 **b** ), and the gastroduodenal artery was embolized by interventional radiology.

**Fig. 1:**
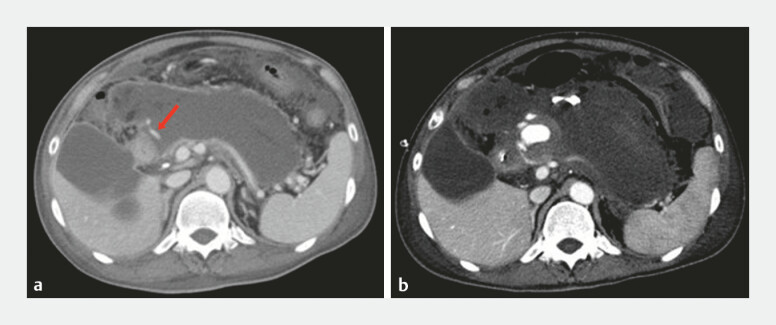
Computed tomography images showing: **a** on admission, the gastroduodenal artery (red arrow) coursing into the walled-off necrosis (WON); **b** 2 days after the transgastric placement of two double-pigtail plastic stents, a pseudoaneurysm originating from the gastroduodenal artery inside the WON.

DEN was started twice weekly from 48 days post-embolization as the necrotic material within the WON remained largely unchanged ( Fig. 2 ). During the seventh and final DEN session, performed 71 days post-embolization, exposed coils were visible within the WON. The coils were not removed owing to concerns about inducing bleeding, as they were firmly attached to the WON wall. The patient was discharged with a debrided WON cavity on hospital day 100 and has remained well for the subsequent 5 years ( Fig. 3 ).

**Fig. 2:**
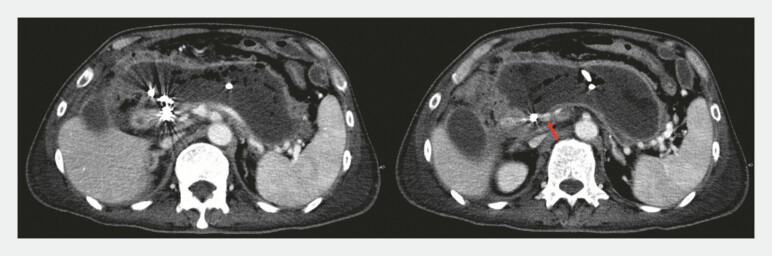
Computed tomography images taken 42 days after transcatheter arterial embolization for a pseudoaneurysm of the gastroduodenal artery inside the walled-off necrosis (WON) showing significant amounts of intra-WON necrotic remnants and blood flow (red arrow) just adjacent to the embolization coils in the wall of the WON.

**Fig. 3:**
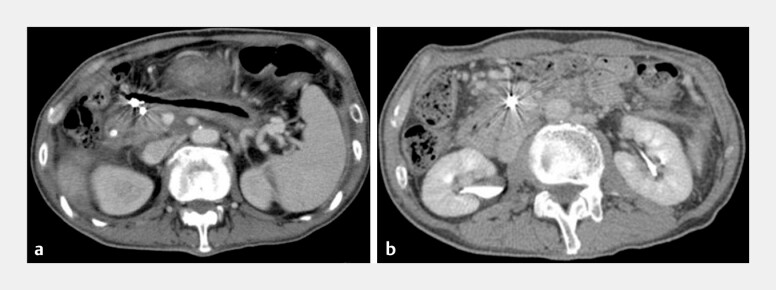
Computed tomography images showing: **a** 2 months after the completion of direct endoscopic necrosectomy (DEN), the embolization coils within the gastroduodenal artery pseudoaneurysm in the debrided walled-off necrosis (WON) cavity; **b** 1 year after the completion of DEN, the coils remaining within the regressed cavity of the WON.

Our case highlights that leaving the embolization coils adherent to the wall inside the WON contributes to preventing bleeding during DEN and might not become a source of infection in the late phase after DEN.

Endoscopy_UCTN_Code_TTT_1AR_2AI

## Notes

Endoscopy E-Videos https://eref.thieme.de/e-videos
*E-Videos* is an open access online section of the journal *Endoscopy* , reporting on interesting cases and new techniques in gastroenterological endoscopy. All papers include a high-quality video and are published with a Creative Commons CC-BY license. Endoscopy E-Videos qualify for HINARI discounts and waivers and eligibility is automatically checked during the submission process. We grant 100% waivers to articles whose corresponding authors are based in Group A countries and 50% waivers to those who are based in Group B countries as classified by Research4Life (see: https://www.research4life.org/access/eligibility/ ). This section has its own submission website at https://mc.manuscriptcentral.com/e-videos .
